# Identifying Age‐Modulating Compounds Using a Novel Computational Framework for Evaluating Transcriptional Age

**DOI:** 10.1111/acel.70075

**Published:** 2025-04-30

**Authors:** Chao Zhang, Nathalie Saurat, Daniela Cornacchia, Sun Young Chung, Trisha Sikder, Adrianne Nemchik, Andrew Minotti, Lorenz Studer, Doron Betel

**Affiliations:** ^1^ Section of Computational Biomedicine Boston University School of Medicine Boston Massachusetts USA; ^2^ Institute for Computational Biomedicine Weill Cornell Medicine New York New York USA; ^3^ Division of Hematology and Medical Oncology, Department of Medicine Weill Cornell Medicine New York New York USA; ^4^ The Center for Stem Cell Biology Sloan‐Kettering Institute for Cancer Research New York New York USA; ^5^ Developmental Biology Program Sloan‐Kettering Institute for Cancer Research New York New York USA; ^6^ Weill Graduate School of Medical Sciences of Cornell University New York New York USA

**Keywords:** age score, aging, Alzheimer's disease, cortical neurons, disease modeling, human pluripotent stem cells, transcriptional age

## Abstract

The differentiation of human pluripotent stem cells (hPSCs) provides access to a wide range of cell types and tissues. However, hPSC‐derived lineages typically represent a fetal stage of development, and methods to expedite the transition to an aged identity to improve modeling of late‐onset disease are limited. In this study, we introduce RNAge, a transcriptome‐based computational platform designed to enable the evaluation of an induced aging or a rejuvenated state. We validated this approach across independent datasets spanning different tissues and species, and show that it can be used to evaluate the effectiveness of existing age‐retaining or age‐modulating interventions. We also used RNAge to perform an in silico compound screen using the LINCS L1000 dataset. This approach led to the identification and experimental confirmation of several novel compounds capable of inducing aging or rejuvenation in primary fibroblasts or hPSC‐derived neurons. Additionally, we observed that applying this novel induced aging strategy to an hPSC model of Alzheimer's disease (AD) accelerated neurodegeneration in a genotype‐specific manner. Our study offers a robust method for quantifying age‐related manipulations and unveils compounds that significantly broaden the toolkit for age‐modifying strategies in hPSC‐derived lineages.

## Graphical abstract

RNAge is a transcriptional based computational approach to measure changes in cellular age upon experimental manipulation. In silico screening of a database of transcriptional changes from drug perturbations identified a novel aging agent that was applied to an hPSC model of neurodegenerative disease to trigger a late‐onset phenotype.

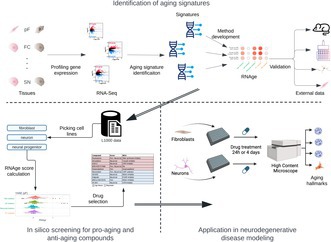

## 1 Introduction

Human PSC‐derived lineages represent fetal rather than adult or aged states (Liu et al. 2012; Mertens et al. 2021; Miller et al. 2013) which poses significant challenges when modeling age‐dependent disorders, where aging increases the penetrance of disease across tissue types. This challenge is especially salient to neurons, where access to primary tissue is limited, and replicative senescence cannot be used to model cellular aging. Accordingly, there is a major interest in developing strategies that can fast‐forward cellular age in hPSC‐derived cells. In neurons, these efforts can be grouped into two broad approaches: induced aging of developmentally specified hPSC‐derived neurons (Fathi et al. 2022; Miller et al. 2013; Vera et al. 2016) versus transdifferentiation of aged fibroblasts directly to neurons (Mertens et al. 2015, 2021). Transdifferentiation of fibroblasts from an aged donor directly to neurons maintains the age‐related signature present in the primary fibroblast. However, this strategy involves several technical challenges including low transdifferentiation efficiency, scalability, and stability of the resulting neurons. Recent studies also indicate that aging may show tissue and cell type specificity (Buckley et al. 2023; Oh et al. 2023) raising the question of how relevant fibroblast aging signatures are to neuronal age when using a transdifferentiation approach. In contrast, induced aging approaches of hPSC‐lineages offer the advantage of scalability, the flexibility to direct cell type specificity, and the use of a broad range of cellular and genetic engineering technologies that are used routinely in the hPSC disease modeling field. However, there is no consensus on the most relevant strategies to trigger aging‐like states in hPSC‐derived lineages.

To compare, evaluate, and expand on any age‐modifying interventions, it is essential to develop methods that quantitatively measure biological age at the cellular level. A commonly used approach is DNA methylation‐related aging clocks that are based on 5mC methylation status at tissue‐ and age‐related CpG sites (Horvath 2013; Horvath and Raj 2018). In addition, there are transcriptomics‐based aging clocks, which offer an obvious advantage due to the broad availability of data sets spanning many cell types, disease conditions, and cellular states. Transcriptomic clocks may also provide more direct mechanistic insights into the aging process than epigenetic measurements. There have been several efforts to develop both global and cell type‐specific transcriptional aging clocks (Buckley et al. 2023; Fleischer et al. 2018; Jung et al. 2023; Lu et al. 2023; Meyer and Schumacher 2021). To date, the use of the various aging clocks has been limited to estimating absolute or relative cellular age. An exciting future application is their use as a direct readout in functional screens aimed at age manipulation to identify novel compounds that enable improved hPSC‐based models of late onset human disease.

Here, we develop RNAge, a novel transcriptomic‐based method that is suitable to routinely score changes in the relative age of human cells. We further used RNAge to identify novel candidate age‐modifying compounds by performing in silico drug screens in the LINCS L1000 database of cellular perturbations (Subramanian et al. 2017) (https://lincsproject.org). Finally, we showed that the age‐inducing compounds identified here can trigger degeneration in neurons carrying an autosomal dominant Alzheimer's disease‐causing mutation while sparing the isogenic control neurons. Our study presents a novel approach for the identification of age‐modulating compounds that may enable the more faithful modeling of late‐onset disease phenotypes in vitro.

## 2 Results

### 2.1 Establishment and Validation of RNA‐Seq‐Based Aging Scores

**FIGURE 1:**
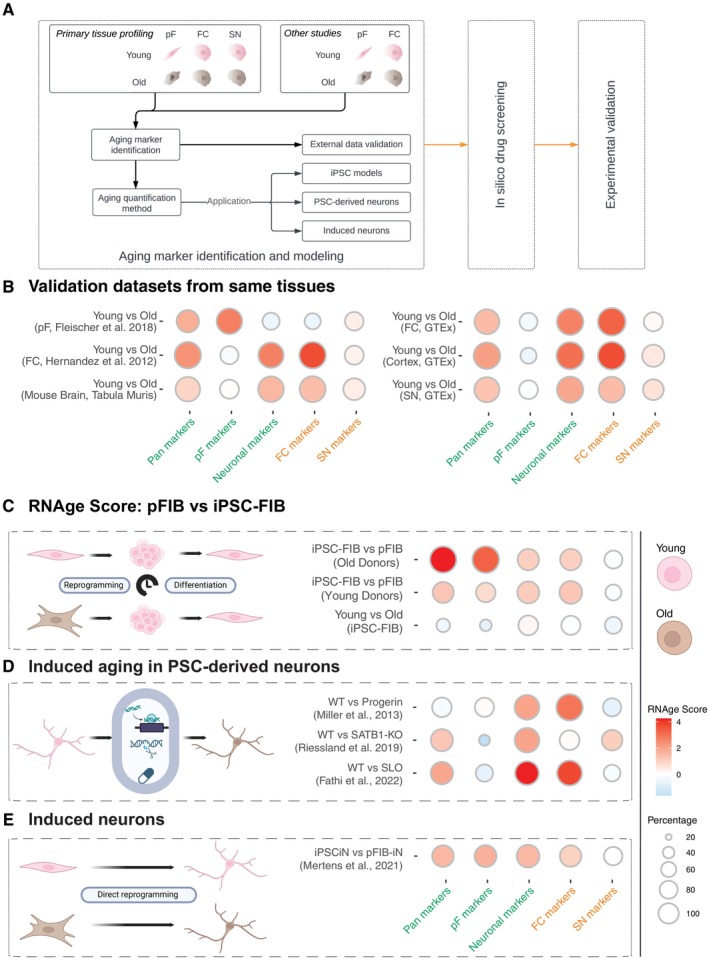
Development of RNAge score and application to stem cell‐based in vitro models. (A) Schematic outline of the workflow to derive, validate and apply RNAge. Cells and tissues used to derive RNAge were pF (primary fibroblasts), FC (frontal cortex), and SN (substantia nigra) samples from young (pink) and old (brown) donors. (B) Validation of the RNAge scores for Pan, pF, FC, Cortex, SN, and mouse brain using external data that was not used to derive RNAge. Tissue type, species, and data source are indicated on the bubble plot. (C) Application of RNAge to measure rejuvenation upon reprogramming of primary fibroblasts to pluripotent stem cells and differentiation to iPSC‐derived fibroblasts. The RNAge score is increased when old pFIB are compared to iPSC‐FIB than when young donors are used. (D) Application of RNAge scoring to established experimental approaches for age induction including ectopic expression of Progerin (Miller et al. 2013), SATB1‐KO (Riessland et al. 2019) and SLO cocktail (Fathi et al. 2022) (E). Application of RNAge to neurons generated by transdifferentiation of primary fibroblasts (pFIB‐iN) relative to neurons generated in the same manner from iPSCs (iPSCiN) (Mertens et al. 2015). In all bubble plots, the primary sub scores indicated in green and secondary sub scores in orange.

To enable the development of stem cell‐based models that combine precise developmental patterning with the ability to specify cellular age, we first developed a tractable and reproducible method of scoring age (Figure 1A). To achieve this, we first identified sets of genes whose expression can be used to distinguish different age groups. Gene sets were established by performing total RNA sequencing of young (7–14 years) and old (70–96 years) primary human tissue samples (fibroblasts, cortex, substantia nigra [SN]; Figure S1A–C, Tables S1 and S2). To increase the robustness of those age‐related signatures, we also incorporated external datasets (Mertens et al. 2015) as well as internal poly‐A RNA‐seq data from additional patient samples. This approach allowed us to identify a set of five mutually exclusive aging signatures: pan‐aging genes that mark aging across all three lineages, fibroblast‐specific, pan‐neuronal, frontal cortex (FC)‐specific, and SN‐specific aging signatures (Figure S1D).

Using six age‐associated datasets, we established a computational pipeline (“RNAge”) to measure expression changes in relative age between two groups of samples (Figure S1E). We ranked the age associated genes based on the combined *p*‐values from all training datasets for each aging gene signature panel (pan‐aging, fibroblast, pan‐neuronal, FC and SN panels) individually. The top 100 ranked signatures from each panel were then used to establish our scores, named ‘Pan,’ ‘pF,’ ‘Neuronal,’ ‘FC,’ and ‘SN’. The aging score is based on both the magnitude of the difference in expression between the two datasets and the directionality of the change across the top 100 age‐regulated genes (see methods and Table S3). We also calculate and visualize the percentage of genes of the age signature that change their expression in the expected direction to assess if the aging score is driven by very large changes in the expression of a small subset of the age‐associated genes or by a more moderate change across most of the genes. (Figure S1E,F). We further sub categorize our aging scores into primary scores which are tissue specific (‘Pan,’ ‘pF,’ and ‘Neuronal’) and secondary scores which reflect region specific changes in the aging CNS (‘FC’ and ‘SN’). Primary scores are indicated in green text and secondary scores in orange text.

As a first step we performed RNAge scoring on the datasets used to develop our transcriptional age score (Figure S1F) and demonstrate the specificity of both the primary and secondary aging scores. To comprehensively validate RNAge, we applied it to independent data sets from the same tissues including transcriptomic datasets from young versus old human primary fibroblasts (Fleischer et al. 2018); in both RNA‐seq and microarray‐based comparisons of young versus old human FC (GTEx Consortium 2013; Hernandez et al. 2012) and young and old mouse brain (Figure 1B). In contrast, RNAge was not applicable to stomach (GTEx Consortium 2013) (Figure S1G) indicating that use of RNAge should be limited to the tissues used to derive the aging signature. Within the CNS, the primary aging scores (pan, fibroblast and neuronal) proved to be broadly applicable across neuronal subtypes, performing consistently in young and old Substantia Nigra, Cerebellum, Hippocampus, and Spinal Cord (GTEx Consortium 2013) (Figure 1B, Figure S1G). While the secondary, regional aging scores provided some differentiation between regions, they were less consistent and most applicable to the FC. This was also the case for the SN, likely due to the availability of only a single dataset for training purposes.

To further test RNAge's applicability, we performed RNAge scoring on glioblastoma (GBM) samples (Ainslie et al. 2024) and found that cancer increased transcriptional age, consistent with previous findings using DNA methylation‐based aging scores (Horvath 2013). This supports the utility of RNAge not only for assessing normal aging processes but also for evaluating the impact of disease on transcriptional age within the CNS.

### 2.2 Measuring Cellular Rejuvenation in Stem Cell Models

Transcriptional reprogramming of primary fibroblasts to pluripotent stem cells is one of the best described methods of resetting cellular age (Lapasset et al. 2011; Miller et al. 2013; Ocampo et al. 2016). As a positive control for RNAge, we reprogrammed a subset of our fibroblast lines to pluripotent stem cells, then differentiated them back into iPSC‐derived fibroblasts (Figure 1C). After confirming that reprogramming and re‐differentiation were successful (Figure S2A, Table S4), we performed RNA‐seq and applied RNAge. Reprogramming fibroblasts to iPSCs resulted in the loss of age‐related differentially expressed genes (DEGs) that were not re‐established once iPSCs were differentiated back into iPSC‐derived fibroblasts (Figure S2B). We then calculated the RNAge score and showed that both young and old primary fibroblasts show an increased aging score relative to iPSC‐derived fibroblasts (Figure 1C). Conversely, there was no difference in the aging score between iPSC‐derived fibroblasts from young and old donors despite the large difference in age of the original donors (Figure 1C). Together, these results further validate the use of the RNAge score to assess age‐related transcriptional changes, support previous findings that aging signatures are erased during reprogramming (Lapasset et al. 2011; Miller et al. 2013), and validate the use of our aging score in measuring cellular rejuvenation.

### 2.3 Evaluating Manipulations That Induce Cellular Age

Age induction strategies are critical for the study of late onset diseases in hPSC‐based models and may open new avenues for identifying and validating novel therapeutic interventions. Therefore, it was important to determine whether RNAge can be used to compare cellular age in existing hPSC‐based induced aging models (Figure 1D). Several distinct age induction strategies have been described for use in hPSC‐derived neurons including: (i) ectopic expression of Progerin (Miller et al. 2013), the protein responsible for the premature aging condition Hutchinson‐Gilford progeria syndrome (HGPS); (ii) the knockout of SATB1 in dopaminergic neurons resulting in increased cellular senescence (Riessland et al. 2019); (iii) chemical induction of senescence in fibroblast and neurons using the “SLO” small molecule cocktail (Fathi et al. 2022); and (iv) the induction of cellular aging hallmarks as a result of impaired function of the neddylation pathway (Saurat et al. 2024). Using our novel aging scoring methods, we observed that the ectopic expression of Progerin in iPSC‐derived neurons triggered a pan ‘Neuronal’ and ‘FC’ aging signature but not an increase in the ‘Pan’ aging score. This is consistent with our results from primary HGPS fibroblasts that did not show a ‘Pan’ aging or ‘pF’ aging score (Figure S2). Previous studies have also shown that SATB1 and the small molecule cocktail SLO can induce cellular aging hallmarks (Fathi et al. 2022; Riessland et al. 2019). Consistent with those findings, we observe that SATB1 KO and SLO treatment induce a robust increase in the transcriptional aging score and the expected subscores (Figure 1D).

### 2.4 RNAge Detects Neuronal and Fibroblast Aging Scores in Directly Reprogrammed Neurons

An alternative approach to incorporate age into stem cell models is to directly convert primary fibroblasts from young and old individuals to induced neurons (iNs) by the forced expression of Ngn2 and Ascl1 (Mertens et al. 2015; Pang et al. 2011; Yoo et al. 2011) or expression of miR‐9/9*‐124 along with NEUROD2 and MYT1L (Sun et al. 2024; Yoo et al. 2011). iNs that are derived from fibroblasts of old individuals retain a transcriptional aging signature and show age‐associated disease phenotypes in models of neurodegeneration (Herdy et al. 2022; Mertens et al. 2021, 2015; Sun et al. 2024). We tested whether iNs generated from old fibroblasts had a higher aging score compared to iNs generated from PSCs (Mertens et al. 2021) where cellular age is reset during reprogramming (Figure 1E). Our aging score showed that forebrain‐like neurons generated from old fibroblasts displayed an increase in the ‘Pan,’ ‘Neuronal,’ and ‘FC’‐specific aging scores but not the ‘SN’ aging score. However, unexpectedly, these fibroblast‐derived neurons also showed expression of a fibroblast‐specific ‘pF’ aging score, suggesting that they maintained age‐related features of the original fibroblast, an aging signature that is not present in neurons that have undergone chronological aging (compare to Figure 1B).

### 2.5 RNAge Score Is Biologically Tractable and Can Be Used to Identify Age‐Regulating Compounds

**FIGURE 2:**
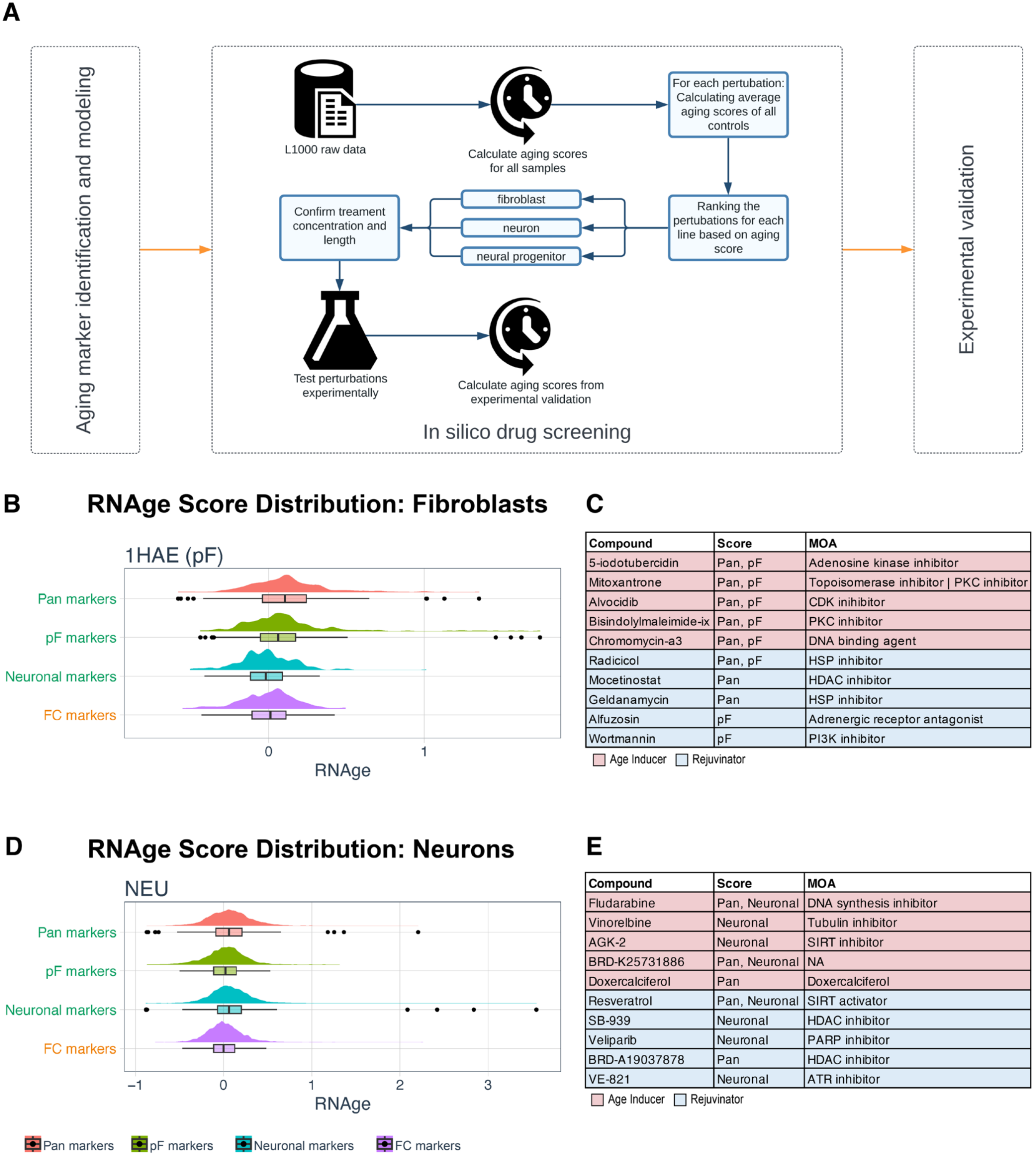
In silico screening for modulators of RNAge. (A) Workflow to use the L1000 dataset for performing in silico drug screening for regulators of RNAge. (B) RNAge score distribution for L1000 in silico screen performed in primary fibroblasts (1HAE). Black dots indicate hits that are > 3 SD from mean and primary sub scores are indicated in green and secondary sub scores in orange. (C) List of top scoring RNAge modifying compounds from (B) and their mechanism of action (MOA). (D) RNAge score distribution for L1000 in silico screen performed in neurons (NEU). Black dots indicate hits that are > 3 SD from mean and primary sub scores are indicated in green and secondary sub scores in orange. (E) List of top scoring RNAge modifying compounds from (D).

Our study demonstrates that RNAge can be used to compare the relative age of primary tissues and hPSC‐derived cells in response to different aging and rejuvenating paradigms. We next wanted to test whether it could also be used as a drug discovery tool to identify tissue‐specific drivers of cellular age. To achieve this goal, we designed an in silico screen (outlined in Figure 2A) using the LINCS L1000 dataset. This is a large dataset of transcriptional changes induced by > 1 million pharmacological compounds or genetic perturbations in a diverse set of cell lines (Subramanian et al. 2017). Given the evidence for cell‐type specificity of the aging signatures presented, we restricted our screen to a smaller subset of perturbations performed in the cell types that are most similar to the primary tissues used to derive our fibroblast and neuronal age signatures (1HAE and NEU). 1HAE is a normal fibroblast line while NEU corresponds to iPSC‐derived neurons. We further restricted our analysis to drug‐based perturbations as these are more amenable for widespread use as a tool in iPSC‐based disease modeling. By restricting our analysis to those parameters, the L1000 dataset included a total of 348 and 3968 perturbations for 1HAE and NEU, respectively. We calculated the aging scores for each of those perturbations relative to matched control samples in the dataset and averaged them to get a single value for each perturbation. Aging scores followed a normal distribution for all cell types and sub‐scores (Figure 2B,D) indicating that most drug treatments did not induce changes in the aging score. However, there were several compounds that appeared to rejuvenate or to induce transcriptional age (> 3 standard deviations [SD] from mean RNAge score) that were selected for further study.

### 2.6 HDAC Inhibitors Induce Rejuvenation

Although the primary goal of our study was to identify age induction strategies due to their potential application in establishing improved models of late onset disease, we also addressed whether our screening strategy can identify candidate rejuvenating drugs. Interestingly, HDAC inhibitors (HDAC‐i) were among the top scoring rejuvenating compounds across both cell types tested (Figure 2C,E). To evaluate the consistency of these findings, we calculated the RNAge scores for all compounds applied to NPCs (neural progenitor cells), an additional CNS relevant cell type (Figure S3A). HDAC‐i were also the top scoring rejuvenating compounds (Neuronal subscore) in this cell type (Figure S3B). To experimentally validate HDAC‐i as a regulator of cellular rejuvenation, we treated aged primary fibroblasts with Mocetinostat for 24 h to maintain consistency with the L1000 data generation. We then profiled the samples by RNA‐seq data to calculate the RNAge score for Mocetinostat relative to the DMSO control. Validation experiments were performed in fibroblasts due to the availability of primary fibroblasts isolated from aged individuals, which is not readily feasible for primary human neurons. Mocetinostat treatment was able to reverse cellular aging in old primary fibroblasts across all RNAge sub scores (Figure S3C). To further investigate this finding, we performed ATAC‐seq and RNAseq on primary young and old fibroblasts to identify the top candidate regulators of age in primary fibroblasts (Figure S3D). This showed motifs for E2F factors, HMGA1, and STAT1 to be differentially accessible in young versus old primary fibroblast (Figure S3D). This family of transcription factors is regulated by HDACs (Brehm et al. 1998; Chueh et al. 2015). Our data indicate that RNAge is suitable to identify candidate rejuvenating factors and provides further impetus to study HDAC‐i as potential compounds to promote transcriptional features of cellular rejuvenation.

### 2.7 Identification of Candidate Age Inducers Using the L1000 Dataset

**FIGURE 3:**
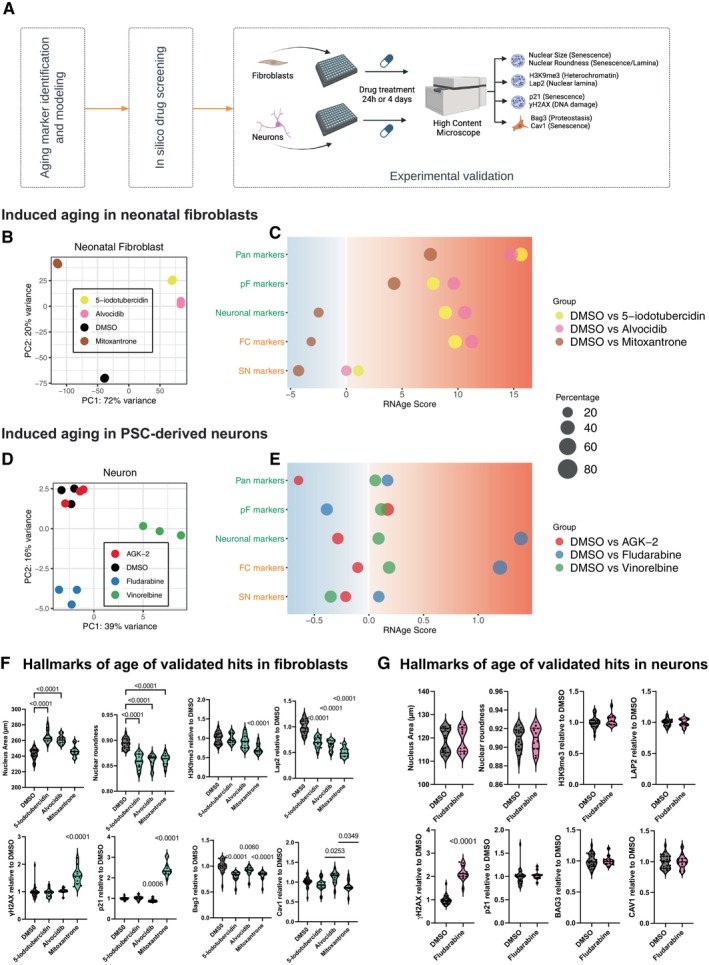
Experimental validation of age inducing compounds identified in silico. (A) Schematic illustration focusing on hit validation from the L1000 screen. Hits were assessed for changes in the cellular hallmarks of aging at 1 day (this figure) or at 4 days (Figure 4A,B) after treatment. (B) PCA of RNA‐seq data from fetal fibroblasts treated with top ranked age inducers identified in the L1000 in silico screen. (C) RNAge subscores for fibroblast treated with age inducers (see (B)). Primary sub scores are in green text and secondary sub scores are in orange text. (D) PCA of RNA‐seq data from young PSC‐derived neurons treated with top ranked age inducers identified in the L1000 in silico screen. (E) RNAge subscores for neurons treated with age inducers (see D). Primary sub scores are in green text and secondary sub scores are in orange text. (F, G) Measurement of the cellular hallmarks of aging age in fibroblasts (F) or neurons (G) in response to compounds validated for inducing transcriptional age shown in (C, E). Absolute values are shown for nuclear area and nuclear roundness. For H3K9me3, LAP2, yH2AX, p21, BAG3, and CAV1, fluorescence intensity is relative to the DMSO control. Experiment was repeated four times each comprising three replicate wells per condition (*n* = 12). For nuclear area and roundness measurements ordinary one‐way ANOVA with Dunnett's multiple comparison test were performed. For intensity measurements one sample *t*‐tests were used.

We next focused on compounds that induced an increase in RNAge in fibroblasts and in neurons for the L1000 dataset (Figures 2B–E and 3A). For experimental validation in fibroblasts, we selected top scoring compounds including alvocidib (CDK inhibitor), 5‐iodotubercin (adenosine kinase inhibitor) and mitoxantrone (Topoisomerase II inhibitor), as these three drugs were among the top four age inducers for both the ‘Pan’ and ‘pF’ aging scores. We selected Fludarabine (STAT1 and DNA synthesis inhibitor), vinorelbine (microtubule antagonist) and AGK‐2 (SIRT2 inhibitor) for further validation in neurons, as these were the top three candidate regulators of the pan‐neuronal score. Fludarabine was also the top candidate as measured by the ‘Pan’ aging score. To select an appropriate dose for experimental validation, we analyzed the L1000 dataset, which included datasets across multiple independent perturbations with varying drug concentrations and treatment lengths for many candidate age regulators (Figure S4A,B).

We treated fetal human dermal fibroblasts (HDF‐f) with all three of our candidate age inducers and performed the polyA selection bulk RNAseq. Principal component analysis (Figure 3B) of our RNA‐seq data indicated that all the fibroblast aging inducers segregated from the control, with Alvocidib and 5‐iodotubercin clustering closely together. Next, we used the RNA‐seq data to calculate the RNAge score for each of the perturbations relative to DMSO (Figure 3C). All three of our candidate drugs resulted in an increased age score, confirming our in silico scoring results (Figure S4A). Alvocidib and 5‐iodotubercin, which clustered together on the PCA plot, resulted in a very similar pattern of induction of the RNAge subscores (Figure 3C, pink and yellow) whereas mitoxantrone was distinct and only showed induced aging in the ‘Pan’ and ‘pF’ aging categories (Figure 3C, brown).

We next treated the PSC‐derived neurons with the corresponding candidate age‐inducing compounds and performed the polyA RNA sequencing. Principal component analysis for the putative inducers of neuronal age suggested that fludarabine and vinorelbine induced distinct transcriptional changes in neurons (Figure 3D). AGK2 treatment had no obvious transcriptional effect or impact on the RNAge score (Figure 3E). Aging score induction in the L1000 dataset was highly concentration‐dependent (Figure S4B), and it is therefore possible that the optimal concentration or treatment differed in our experimental system. Vinorelbine‐treated neurons showed only a slight, albeit consistent, induction of aging scores across the aging signatures (Figure 3E, green). In contrast, Fludarabine strongly induced aging in neurons based on both the ‘Neuronal’ and ‘FC’ subscores and showed a weak induction of the ‘Pan’ aging score (Figure 3E, blue). These results indicate that fludarabine may be an effective tool to induce age‐like transcriptional states in young hPSC‐derived neurons.

### 2.8 Correlation of RNAge With Hallmarks of Cellular Age

Next, we wanted to assess whether the age‐modifying drug treatments also trigger the well‐described cellular hallmarks of age (Lapasset et al. 2011; Miller et al. 2013) (Figure 3A) including loss of heterochromatin (H3K9me3 and LAP2), disrupted nuclear lamina (decreased nuclear roundness and loss of LAP2), increased DNA damage (γH2AX), increased cellular senescence (p21 and nuclear area), disrupted proteostasis with increased dependence on autophagy (BAG3, Gamerdinger et al. 2009; Klimek et al. 2017; neuronal hallmark), and increased CAV1 (multiple functions, broadly increased with age, Ha et al. 2021; Kang et al. 2006; Volonte et al. 2002; Wheaton et al. 2001). These aging hallmarks encompass many of the hallmarks and assays used to measure cellular senescence, which is an integral feature of cellular aging (López‐Otín et al. 2013, 2023).

Alvocidib, 5‐iodotubercin, and mitoxantrone (24 h) all increased the relative transcriptional age of fetal fibroblasts (Figure 3C). We observed that all those interventions induced a loss of nuclear roundness and loss of LAP2, suggesting that nuclear lamina defects are a conserved feature of increased cellular age in fibroblasts. All interventions also resulted in a decrease in BAG3 (Figure 3F, Figure S4C). Treatment with alvocidib and 5‐iodotubercin also induced an increase in nuclear area (Figure 3F). In contrast, mitoxantrone treatment increased γH2AX and p21 and decreased H3K9me3 immunoreactivity (Figure 3F). This subset of hallmarks is strongly associated with cellular senescence (de Luzy et al. 2024; Carlos López‐Otín et al. 2013; C. López‐Otín et al. 2023). These results indicate that within a specific cell type, our aging score can be associated with distinct subsets of the aging hallmarks. In neurons, 24 h of fludarabine treatment was experimentally validated to induce a transcriptional age (Figure 3E). However, the DNA damage marker γH2AX was the only cellular hallmark of age that changed in response to this treatment at the 24 h time point (Figure 3G, Figure S4D).

### 2.9 Time Dependent Induction of Cellular Hallmarks of Aging

**FIGURE 4:**
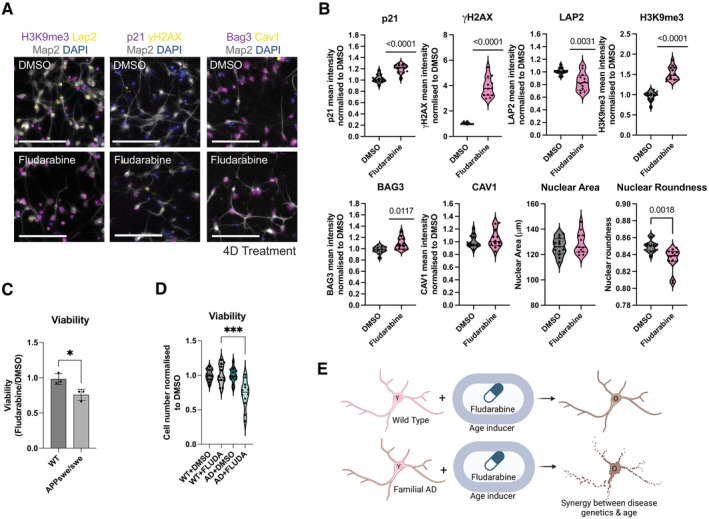
Incorporating induced aging into stem cell models of late onset disease. (A) Representative immunocytochemistry images for the hallmarks of aging assays in PSC‐derived neurons after 4 days of treatment with fludarabine. Scale 100 μm. (B) Quantification of the cellular hallmarks of aging in WT neurons after 4 days of treatment with fludarabine. Absolute values are shown for nuclear area and nuclear roundness and significance testing performed using unpaired *t*‐tests. For H3K9me3, LAP2, γH2AX, p21, BAG3, and CAV1, fluorescence intensity is relative to the DMSO control. *N* = 12; *p* values are calculated using a one sample *t*‐test. Experiment was repeated four times each with three replicate wells. (C) Presto blue viability assay in WT and APPswe/swe neurons treated with fludarabine for 4 days relative to DMSO treatment (*n* = 3, paired Student's *t*‐test). (D) Total cell neuron number for WT and AD (APPswe/swe) neurons treated with DMSO or Fludarabine. Cell number was normalized to the average cell number for the DMSO controls for each differentiation (*n* = 12; Experiment was repeated 4 times each with 3 replicate wells and the unpaired *t*‐test was used to compare the viability of fludarabine treated neurons in WT and AD. (E) Schema outlining how fludarabine induced aging can synergize with AD‐causing mutations to induce neuronal loss.

It was not clear whether the limited induction of cellular aging hallmarks upon fludarabine treatment was because the transcriptional aging in neurons is decoupled from the induction of the canonical hallmarks of cellular age or because transcriptional changes precede the widespread induction of the cellular aging hallmarks. To test this, we extended fludarabine treatment from 24 h to 4 days and repeated our analysis (Figure 4A,B). Under these conditions, yH2AX was further induced and several additional canonical hallmarks of aging were also induced including p21 immunoreactivity, loss of LAP2, increased BAG3, and a decrease in nuclear roundness. However, CAV1 and nuclear area were unchanged whereas H3K9me3 was increased rather than decreased. Although H3K9me3 loss is traditionally considered a hallmark of aging, it can also increase in aged cells due to the formation of senescence‐associated heterochromatin foci (SAHF), which are induced by persistent and high levels of DNA damage (Narita et al. 2003; González‐Gualda et al. 2021). Since SAHF formation could confound our ability to distinguish whether H3K9me3 changes were due to heterochromatin loss associated with aging or SAHF, we tested a lower concentration of fludarabine (1 μM) to reduce persistent DNA damage and minimize SAHF formation. Under these conditions, we observed a smaller but significant increase in DNA damage and a significant reduction in H3K9me3, consistent with heterochromatin loss during aging (Figure S5A).

### 2.10 Effects of Fludarabine on Aging of Dopaminergic Neurons

To determine whether fludarabine could be used to induce aging in other neuron subtypes, we differentiated PSCs to dopaminergic neurons (Kim et al. 2025), treated them with 10 μM fludarabine for 4 days, and assessed the hallmarks of aging by immunohistochemistry. fludarabine treatment induced DNA damage, as evidenced by increased pATM staining, and elevated the senescence‐associated marker p21. However, other aging hallmarks observed in cortical neurons, such as chromatin remodeling and proteostasis disruption, were not detected in dopaminergic neurons (Figure S5B).

### 2.11 Using Novel Aging Strategies to Model Late‐Onset Disease

A key application of induced aging strategies is their incorporation into PSC‐based models of disease to enable the study of late onset phenotypes in a dish. Traditional PSC‐derived models of AD are effective at modeling changes in APP processing that are associated with early onset genetic forms of AD, but modeling the neurodegenerative phase of the disease has been challenging. To test whether age induction in an AD genetic model can mimic the neurodegenerative phase, we differentiated an isogenic pair of hPSC lines edited to carry the APPswe/swe mutation (Saurat et al. 2024) into cortical neurons. After 4 days of treatment with Fludarabine, the time point where we observed widespread induction of the hallmarks of age (Figure 4A,B), we noticed an AD‐specific loss of neuronal viability relative to the DMSO control. We quantified those findings using the presto blue viability assay (Figure 4C) as well as quantification of cell number by high‐content imaging (Figure 4D). This effect was specific to fludarabine and not seen with the commonly used DNA damage inducer bleomycin (Figure S5C,D). Our results suggest that AD genetic susceptibility can synergize with fludarabine‐mediated induction of cellular age to trigger genotype‐specific neuronal loss, a phenotype that cannot be captured in most hPSC‐based in vitro models of AD (Figure 4E).

## 3 Discussion

Here we present a novel transcriptomic measure of relative age and demonstrate its utility in benchmarking published age‐inducing strategies and identifying novel age‐modifying strategies. Interestingly, aging scores showed tissue specificity suggesting that transcriptional aging, and perhaps the aging process more generally, may be tissue dependent. This idea is supported by proteomics‐based clocks (Oh et al. 2023) and by a recent study by Buckley et al. (2023) who developed and validated cell type‐specific aging clocks and showed that the different cell types in the mouse SVZ respond differently to known rejuvenation strategies. However, the transcriptomic clock developed by Buckley et al. was not suitable for our application as it focuses on proliferating neural progenitors and does not capture neuronal aging. Furthermore, the study was limited to the mouse and may not be directly applicable to the human CNS given the prominent species‐specific differences in SVZ biology (Sanai et al. 2004). In contrast, Jung et al. demonstrated that it is possible to generate a highly predictive RNA‐seq‐based human aging clock that can be used universally across tissue types (Jung et al. 2023). However, this method showed limited success in predicting cellular age in response to known age‐related interventions, such as metformin, rapamycin, and hypoxia. This result may indicate that those interventions do not, in fact, increase relative age. Alternatively, it may suggest a lack of sensitivity for a universal aging clock in detecting age‐related perturbations, given our evidence for cell‐ and tissue‐specificity of aging mechanisms.

Age is the strongest risk factor for several neurodegenerative diseases, and there is evidence that cellular age can also impact disease progression (Fathi et al. 2022; Herdy et al. 2022; Mertens et al. 2021; Miller et al. 2013; Vera et al. 2016). This study identified fludarabine as a regulator of transcriptional age in neurons, and we demonstrate that fludarabine can trigger neuronal loss in a genotype‐specific manner. Therefore, fludarabine presents a novel strategy to trigger age‐related disease phenotypes in a genetic model of AD similar to our recent work on inhibiting neddylation using genetic or pharmacological strategies (Saurat et al. 2024). In addition, fludarabine was able to induce hallmarks of senescence in dopaminergic neurons, suggesting that it may also be useful in studying PD in vitro. However, while the work on neddylation inhibition was based on a primary genetic screen for disease modification, in the case of fludarabine, robust disease modification was observed even though the primary screen was based on an in silico screen using transcriptional aging signatures as a probe set. This suggests that the in silico screening approach can be an effective strategy to identify age‐inducing and potential cell rejuvenating compounds and joins other strategies for inducing cellular aging such as mimicking aging triggered in progeroid syndromes, senescence‐based aging screens, the targeting of selective hallmarks of age such as telomere function, or the use of disease modification screens.

We observed robust transcriptional rejuvenation signatures when comparing old primary fibroblasts with their matched iPSC‐derived fibroblast counterparts, in agreement with past studies that focused on the rejuvenation of cellular hallmarks of age (Lapasset et al. 2011; Miller et al. 2013). Using the L1000 dataset, we also identified HDAC inhibitors as a class of small molecules that were able to reset cellular age. HDAC‐i are one of the compounds that have been incorporated into chemical‐based partial reprogramming strategies (Schoenfeldt et al. 2022; Yang et al. 2023) and have been shown to improve age‐related healthspan (Tammaro et al. 2024) and late‐onset disease phenotypes in mouse models (McIntyre et al. 2019), as well as lifespan in some age‐related models in vivo (Krishnan et al. 2011; Zhao et al. 2005). Further supporting the role of HDAC inhibitors in rejuvenation, we observed differential accessibility of transcription factor motifs, including the E2F family, HMGA1, and STAT1, between young and old fibroblasts in our ATAC‐seq analysis. These transcription factors are well‐documented to be regulated by HDAC activity (Brehm et al. 1998; Chueh et al. 2015) providing a possible mechanistic link between HDAC activity and transcriptional age. Together, these findings suggest that the rejuvenation potential of HDAC‐i should be explored more across lineages in future studies and act as a proof of principle that RNAge could be a useful tool to identify or optimize additional cellular rejuvenation strategies towards the end goal of identifying new strategies for organ‐specific rejuvenation and repair. In future studies, RNAge should be particularly useful in tracking the dynamics of cell fate rejuvenation versus fate reprogramming when optimizing partial reprogramming strategies and assessing therapeutic strategies for age‐associated diseases.

Our study demonstrates the use of aging scores to perform an in silico screen for novel age‐inducing perturbations. While our current analysis was limited to the L1000 dataset, a similar strategy could be used to probe any other pharmacological perturbation dataset with transcriptomic readouts such as Drug‐Seq (Li et al. 2022; Ye et al. 2018) or PLATE‐Seq (Bush et al. 2017) or genetic perturbation data including possibly single cell‐based strategies such as Perturb‐Seq (Adamson et al. 2016; Dixit et al. 2016), CRISPR‐Seq (Jaitin et al. 2016) or CROP‐Seq (Datlinger et al. 2017). The hits identified using these strategies in conjunction with the L1000 dataset had a high experimental validation rate indicating broad applicability for finding additional age‐related regulators in the future. The performance of novel age inducers can be directly compared using the RNAge score and tested in conjunction with previously published strategies to establish an optimal strategy to induce aging in PSC‐derived neurons. One important question is whether various induced aging strategies act independently and potentially in an additive manner, or whether those strategies converge on inducing a shared aging program. Another interesting avenue to explore will be the maintenance of both a neuronal and fibroblast aging signature upon transdifferentiation of old fibroblasts directly into neurons (iNs). Future studies will have to address to what extent a persistent fibroblast aging signature may impact the use of iNs in modeling neurological disorders and whether persistence of fibroblast age is observed across various iN induction paradigms and over prolonged iN culture periods.

Our study demonstrates that increased transcriptional age is associated with the induction of canonical aging hallmarks in fibroblasts, whereas in neurons, the induction of those hallmarks was limited to increased DNA damage upon short‐term treatment. Nevertheless, a wider subset of the hallmarks of aging in neurons was induced with more prolonged treatment. This suggests that DNA damage may act as the primary driver of age in this specific context and may indicate that RNAge can serve as an early marker of age modification. Therefore, RNAge scoring may facilitate the identification of hallmarks of age that act as drivers of cellular age in different cell types and conditions. We further show that the induction of transcriptional age in fibroblasts can be associated with two distinct patterns of cellular hallmarks of age. Mitoxantrone showed a strong cellular senescence phenotype with increased p21 expression, increased DNA damage, and loss of H3K9me3 in contrast to alvocidib and 5‐iodotubercin. This indicates that the RNAge score can capture aging triggered by distinct cellular mechanisms. In addition, it may be important to determine whether these compounds can also alter cellular age as determined by other modalities such as epigenetic aging clocks (Lu et al. 2023). However, this may pose challenges since DNA methylation clocks have primarily been developed using blood or saliva and are heavily impacted by changes in cell composition, rendering them difficult to use in cell culture models (Z. Zhang et al. 2024).

Human PSCs have become an increasingly important tool for the study of human aging and rejuvenation. Our study presents a simple strategy to score manipulations aimed at inducing, reversing, or retaining cellular age across several experimental paradigms. The identification of novel age‐inducing and rejuvenating compounds enhances the available toolbox in the field for directing cellular age on demand and should contribute to the development of more faithful models of late‐onset disease.

### 3.1 Limitations of This Study

The primary transcriptional aging signatures for pan‐tissue, fibroblast, and neuronal aging (pan, fibs, neu) are extensively validated with external datasets and across multiple platforms, including primary tissue, iPSC‐derived cultures, and direct differentiation models. However, transcriptional age is highly tissue‐specific, and researchers should independently validate the use of these primary signatures when applying them outside the tissues and models examined in this study.

While our scoring approach focused on pan‐tissue, brain, and fibroblast signatures, the same principle can be applied to regional specificity. To explore this, we developed secondary aging signatures for the FC and SN. Within our primary data, these secondary scores were able to show some contrast between regional tissues. However, when tested on external datasets, the regional signatures performed inconsistently, likely due to limited datasets available to generate the regional scores. As more high‐quality datasets become available, our scoring approach could be further refined to enhance the regional specificity of RNAge. Alternatively, the RNAge framework could serve as a template for researchers to develop transcriptional aging scores tailored to specific tissues beyond fibroblasts and the central nervous system. These efforts could extend the utility of RNAge to a broader range of tissues and experimental contexts.

## 4 Experimental Procedures

### 4.1 Resource Availability

#### 4.1.1 Lead Contact

Further information and requests for resources and reagents should be directed to and will be fulfilled by the lead contact, Lorenz Studer (studerl@mskcc.org).

#### 4.1.2 Materials Availability

No new pluripotent stem cell lines were generated for this study. Primary Fibroblast Cells Can Be Obtained From Coriell (see Table S1).

#### 4.1.3 Data and Code Availability

- The sequencing data generated in this study are available at NCBI Sequence Read Archive (https://www.ncbi.nlm.nih.gov/sra) under accessions SRA: PRJNA1107241 and PRJNA1002866.
- Source code files for generating figures are available at: https://github.com/zhangch/RNAgePaper.
- Requests for additional information should be directed to Doron Betel (dob2014@med.cornell.edu) and Chao Zhang (chz2009@bu.edu).

### 4.2 Reprogramming of Primary Fibroblasts to iPSCs

Young and old primary fibroblasts were obtained from Coriell and maintained in α‐MEM + 15% FBS. Fibroblasts were reprogrammed to iPSCs using CytoTune Sendai viruses expressing SOX2, OCT4, KLF4, and c‐MYC as previously described (Fusaki et al. 2009; Miller et al. 2013). After the formation of iPSC colonies (aprox. 30 days after transduction), individual colonies were manually isolated, replated onto MEFs, and maintained in KSR media.

### 4.3 Differentiation of iPSCs to Fibroblasts

Differentiation of iPSCs into fibroblasts was performed as previously described (Miller et al. 2013). In brief, pluripotent stem cells were cultured on MEFs in KSR‐based media supplemented with 10 ng/mL FGF2. For differentiation, colonies were dissociated from the feeder layer using dispase and replated onto gelatin‐coated plates. Cells were maintained in DMEM + 20% heat‐inactivated fibroblasts for 25 days with passaging every 5–6 days. Fibroblasts were isolated by flow cytometry (CD‐13^high^ and HLA‐CD44^high^) after 25 days of culture and cultured for an additional 7 days before harvest.

### 4.4 Fibroblast Maintenance and Culture

Fibroblasts were maintained in DMEM‐F12 containing GlutaMAX, 15% heat inactivated FBS, and 1X Pen/Strep, and they were passaged using trypsin. For validation of L1000 aging score by RNA‐seq, old (GM04204; Coriell) or young (HDF‐f; ScienCell #2300) fibroblasts were plated at a density of 4000 cm^2^. Test compounds were applied when cultures reached 70% confluence.

### 4.5 Generation of Human Cortical Neurons

Cortical neurons were generated by directed differentiation of human embryonic stem cells (H9; WA‐09) maintained in E8 on vitronectin coated plates (Ciceri et al. 2024). For the APP^Swe^ studies, we used isogenic H9 cell lines engineered to carry the disease‐related mutation as described previously (Saurat et al. 2024). Differentiation was performed by seeding pluripotent stem cells onto matrigel coated dishes at a density of 300,000/cm^2^ in the presence of Y27632 (10 μM). After 24 h, the E8 medium was replaced with E6 containing SB431542 (10 μM), LDN193189 (100 nM) and XAV939 (2 μM). After 3 days the XAV939 was removed from the differentiation media, and cells were cultured for an additional 7 days in E6 containing SB431542 (10 μM), LDN193189 (100 nM). The neuroepithelium was maintained for an additional 10 days in neurobasal supplemented with N2 and B27. Media were changed daily throughout the differentiation. At DIV 20, the cultures were dissociated using Accutase to generate a single‐cell suspension and plated out for subsequent experiments in neurobasal medium supplemented with B27, L‐glutamine, BDNF, cAMP, ascorbic acid, and GDNF (neural maintenance media). DAPT was also added to the culture medium from DIV20 to DIV30.

### 4.6 Generation of Human Dopaminergic Neurons

Dopaminergic neurons were generated by directed differentiation of human embryonic stem cells (H9; WA‐09) according to our previously published protocol (Kim et al. 2025). In brief, pluripotent stem cells were seeded onto Geltrex coated dishes at a density of 400,000/cm^2^ in the presence of Y27632 (10 μM). After 24 h, the E8 medium was replaced with Neurobasal supplemented with N2, B27, and L‐glutamine containing SB431542 (10 μM), LDN193189 (250 nM) CHIR99021 (1 μM) and SHH (500 ng/mL). Media were replaced daily for a total of 4 days (from DIV 0 to DIV 3) then the CHIR concentration was increased to 6 μM from DIV 4–DIV 6. Following this, SB431542, LDN193189, and SHH were withdrawn, and cells were cultured for an additional 3 days with only CHIR99021 (6 μM). On DIV 10, media were changed to Neurobasal/B27/L‐glutamine supplemented with CHIR (3 μM), BDNF, cAMP, ascorbic acid and GDNF, TGFβ3 (DA neuron maintenance media). The following day, the monolayer was dissociated with accutase and replated at a density of 1,000,000/cm. Cells were cultured in mDA differentiation with medium consisting of NB/B27/L‐Glu, BDNF, ascorbic acid, GDNF, dbcAMP, and TGFβ3 with 1 μM IWP2 and 100 μg/mL FGF18 added DIV12–DIV16. On Day 16, cells were dissociated with Accutase and replated using the same conditions as DIV11. Cells were cultured in mDA differentiation media + 10 μM DAPT until DIV25. At DIV 25, dopaminergic neurons were purified using MACs as previously described (Kim et al. 2023) and neurons were plated out in 96 well plates to perform immunocytochemistry experiments.

### 4.7 Compounds for Experimental Validation of L1000‐Based Score

All validation experiments were performed after 24 h of compound treatment. For rejuvenation experiments in fibroblasts, Mocetinostat (10 μM; Selleck S1396), Resveratrol (0.37 μM; Selleck S1396) and Radicicol (1.1 μM; Tocris 1589), DMSO (1:1000) and EtOH (1:1000; vehicle control for Radicicol) were applied to old primary fibroblasts (Coriell GM4204). Age induction experiments in young fibroblasts (HDF‐f; ScienCell #2300) were performed with 5‐Iodotubercin (10 μM; Selleck S8314), Alvocidib (10 μM; Selleck S1230) and Mitoxantrone (1.1 μM; Selleck S2485) with DMSO (1:1000) used as a control. Compounds used for age induction experiments in PSC‐derived neurons werefludarabine (10 μM; Selleck S1491) Vinorelbine (10 μM; sc‐205885) and AGK‐2 (0.04 μM; Selleck S7577) with DMSO (1:1000) used as a control.

### 4.8 Assaying Hallmarks of Age by High Content Microscopy

HDF fibroblasts were plated at a density of 4500/cm^2^ and cultured for 48 h before drug treatment. Neurons were plated at a density of 100,000/cm^2^ and cultured for 12 days before the application of candidate age inducers. After 24 h of treatment, culture wells were washed 2× in PBS and then fixed with 4% paraformaldehyde. For immunocytochemistry, cells were permeabilized in PBS + 0.3% Triton and blocked in 5% normal goat serum. All primary antibody incubations were performed overnight at 4° and secondary antibodies at RT for 1 h. Primary antibodies used in this study were: BAG3 (abcam; ab47124), CAV1 (Thermo; MA3‐600), H3K9me3 (abcam; ab176916), LAP2 (BD Bioscience; 611000), p21 (CST; 2947), yH2AX (EMD Millipore; 05‐636), pATM (Thermo; MA1‐2020). Immunocytochemistry images were acquired using an Operetta (PerkinElmer, Waltham, MA) microscope and quantified using the harmony high content analysis software. For both fibroblasts and neurons, 4 independent batches of cells were assayed in triplicate (*n* = 12). Fluorescence intensity was normalized to DMSO, and ordinary one‐way ANOVA was used to compare test conditions to the DMSO control (Prism 9.5.1).

### 4.9 Viability Assay

To measure neuronal viability, PrestoBlue Cell viability Reagent was diluted 1:10. A total of 85 μL of diluted reagent was applied to each 96‐well, incubated for 2 h at 37°, and then assayed. Each well was normalized to the mean absorbance of the DMSO control for each differentiation, and technical replicates were averaged to give a single value for each differentiation.

### 4.10 RNA Extraction

Cells were harvested, lysed, and stored in Trizol (Life Technologies) until further processing. For primary FC or SN, a total of 10 mg of brain tissue was lysed in Trizol aided by a tissue dounce. For experimental validation, L1000 compounds RNA was extracted by the core using the miRneasy Micro Kit (QIAGEN 1071023). In all other cases, RNA was extracted using the Zymo Direct‐zol RNA Microprep Kit according to the manufacturer's instructions.

### 4.11 RNA‐Seq Analysis

Reads were aligned to the hg19 human transcripts using STAR (Dobin et al. 2013) (version 2.5.21b) using default parameters and resulting bam files were sorted and indexed using samtools. Gene counts were obtained using featureCounts (Liao et al. 2014) (version 1.4.3) from sorted bam files using uniquely mapped reads. Genes with no expression counts in any sample were discarded. Differential gene expression analysis was performed using DESeq2 (Love et al. 2014) R package that normalize gene count data to transcription per million (TPM), and then detect differentially expressed genes (DEG) between Young and Old groups with (FDR < 0.1).

### 4.12 ATAC‐Seq

Raw sequencing data were processed using the ENCODE ATAC‐seq pipeline (https://github.com/ENCODE‐DCC/atac‐seq‐pipeline). Briefly, reads were trimmed, filtered, and aligned against the hg19 reference genome using Bowtie2. PCR duplicates and reads mapped to the mitochondrial chromosome or repeated regions were removed. To correct for the Tn5 transposase insertion bias, mapped reads were shifted by +4/−5 base pairs. Peak calling was performed using MACS2 (Y. Zhang et al. 2008), with a *p*‐value cut‐off of < 0.01. Peak‐gene links were established based on the correlation between gene expression from RNA‐seq and chromatin accessibility from ATAC‐seq within ± 0.5 Mb (Corces et al. 2018), considering only positively correlated peak‐gene links for subsequent analysis. To identify potential known motifs associated with ATAC‐seq peaks, chromVAR was used to quantify motif accessibility deviations across all samples (Schep et al. 2017), ranking them based on the correlation between chromVAR motif deviations and gene expression. By integrating the DEGs and the differentially binding peaks, potential epigenetic drivers that regulate gene expression changes during aging were identified.

### 4.13 RNAge Training Datasets

Total pF: Primary fibroblast samples from 9 young (7–14 years old) and 9 old (70–96 years old) profiled by total‐RNA‐seq protocol.

PolyA pF: Randomly picked 4 young (10–11 years old) and 4 old (71–96 years old) out of 18 primary fibroblast samples. These samples were profiled by polyA RNA‐seq kit.

PolyA Gage pF: Primary fibroblast RNA‐seq data from 6 young (< 15‐year‐old) and 4 old (> 70‐year‐old) were downloaded from the E‐MTAB‐3037 dataset (Mertens et al. 2015). All raw data were processed by our RNA‐seq analysis protocol.

PolyA Gage FC: FC RNA‐seq data from four young (< 15‐year‐old) and 4 old (> 70‐year‐old) were downloaded from E‐MTAB‐3037 dataset (Mertens et al. 2015). All raw data were processed by our RNA‐seq analysis protocol.

Total FC: Primary FC samples from nine young (13–14 years old) and nine old (70–91 years old) profiled by total‐RNA‐seq protocol.

Total SN: Primary SN samples from 10 young (13–14 years old) and 9 old (70–91 years old) profiled by total‐RNA‐seq protocol.

### 4.14 Other Datasets

GSE113957: Fleischer et al. collected fibroblast samples from 133 healthy individuals (Fleischer et al. 2018). The FPKM of gene expression was downloaded from the GEO database. Out of 133 samples, 10 young (8–13 years old), 10 old (> 89‐year‐old) and 10 HGPS (2–8 years old) samples were used in this study.

GSE36192: Microarray data of the cerebellum and FC from 396 subjects (total 911 tissue samples) were generated by the North American Brain Expression Consortium (Hernandez et al. 2012). The normalized gene expression matrix was downloaded from the GEO database. Both cerebellum and FC from the same 18 young (10–15 years old) and 16 old (> 92‐year‐old) samples were used in this study.

GSE52431: The raw RNA‐seq data from four iPSC‐derived dopamine neurons with overexpressing Progerin and four corresponding control samples were downloaded and processed with the proposed pipeline (Miller et al. 2013).

GSE132040: Bulk RNA‐seq data from the Tabula Muris Consortium study were downloaded and processed. The whole brain data from young (< 3‐month) and old (> 24‐month) mice were used in this study (Tabula Muris Senis Consortium 2020).

GSE141028: Fathi et al. presented a study of inducing senescence by treating human embryonic stem cells with compounds (Fathi et al. 2022). The raw RNA‐seq data from three treatment and three WT cell lines were downloaded, processed, and used in this study.

E‐MTAB‐5965: Genetic ablation of SATB1 induces a senescence phenotype in human embryonic stem cell (hESC)‐derived DA neurons (Riessland et al. 2019). The raw RNA‐seq data from 3 STAB1‐KO and 3 WT cell lines were downloaded, processed, and used in this study.

E‐MTAB‐10352: The raw RNA‐seq data from 42 samples were downloaded, processed, and used in this study (Mertens et al. 2021), including 17 samples from iPSC derived induced neurons and 25 samples from the direct conversion of human fibroblasts into induced neurons.

GTEx: The FPKM data of four tissues was downloaded from GTEx Portal (GTEx Consortium 2013), including FC, cortex, SN and stomach.

### 4.15 Aging Markers Identification

We derived the aging signature for the different tissue types using the following six datasets:

$$\begin{array}{c} D=\{\text{Total}\_\mathrm{pF}\text{PolyA}\_\mathrm{pF}\text{PolyA}\_\text{Gage}\_\mathrm{pF}\text{Total}\_\mathrm{FC}\\ \;\text{PolyA}\_\text{Gage}\_\mathrm{FC}\text{Total}\_\mathrm{SN}\} \end{array}$$

Differential gene expression analysis was performed using DESeq2 between young and old samples in each of the RNA‐seq datasets, resulting in log fold change values and *p*‐value from each of the six datasets.

- The log2 based fold change between young and old groups for each gene *i* in each dataset *j* is denoted as $L_{i,j}$, where i∈G,j∈D.
- The *p*‐value between young and old groups for each gene in each dataset is denoted as $P_{i,j}$, where i∈G,j∈D.

We discretized the log2 fold change of the genes to indicate the regulatory direction in aging. We defined a simplified log2 fold change as follows:

L¯i,j=01−1if−0.1<Li,j<0.1ifLi,j≥0.1ifLi,j≤−0.1i∈G,j∈D

We combined *p*‐values from all six datasets for each gene with Edgington's method(Heard and Rubi‐nDelanchy 2017),

$$C_{i}=\text{Edgington}\left(P_{i,j}\right)\;i\in G,j\in D.$$

Based on the simplified ${\underline{L}}_{i,j}$, we assign genes to discrete and mutually exclusive aging signatures in hierarchical scheme as follows:

1. $G_{1}=\left\{\mathrm{Pan}\;\text{aging gene}\;i\right\}\;\text{if}\;\mathrm{abs}\left(\sum_{j\in D}{\bar{L}}_{i,j}\right)=6,\;i\in G$
2. $G_{2}=\left\{\text{Fibroblast aging gene}\;i\right\}\;\text{if}\;\mathrm{abs}\left(\sum_{j}{\bar{L}}_{i,j}\right)=3$
$$\text{where}\;i\in \left(G\cap \bar{G_{1}}\right),j\in \left\{\text{Total}\_\mathrm{pF},\text{PolyA}\_\mathrm{pF},\text{PolyA}\_\text{Gage}\_\mathrm{pF}\right\}$$
3. $G_{3}=\left\{\mathrm{Pan}\;\text{neuronal aging gene}\;i\right\}\;\text{if}\;\mathrm{abs}\left(\sum_{j}{\bar{L}}_{i,j}\right)=3$
$$\text{where}\;i\in \left(G\cap \bar{G_{1}}\cap \bar{G_{2}}\right),j\in \left\{\text{Total}\_\mathrm{FC},\text{PolyA}\_\text{Gage}\_\mathrm{FC},\text{Total}\_\mathrm{SN}\right\}$$
4. $G_{4}=\left\{\mathrm{FC}\;\text{aging gene}\;i\right\}\;\text{if}\;\mathrm{abs}\left(\sum_{j}{\bar{L}}_{i,j}\right)=2$
$$\text{where}\;i\in \left(G\cap \bar{G_{1}}\cap \bar{G_{2}}\cap \bar{G_{3}}\right),j\in \left\{\text{Total}\_\mathrm{FC},\text{PolyA}\_\text{Gage}\_\mathrm{FC}\right\}$$
5. $G_{5}=\left\{\mathrm{SN}\;\text{aging genes}\;i\right\}\;\text{if}\;{\bar{L}}_{i,j}\neq 0$
$$\text{where}\;i\in \left(G\cap \bar{G_{1}}\cap \bar{G_{2}}\cap \bar{G_{3}}\cap \bar{G_{4}}\right),j\in \left\{\text{Total}\_\mathrm{SN}\right\}$$

For each aging signature set, we ranked genes based on their combined *p* value $C_{i}$. Thus top 100 genes with smallest *p* values from each subset were selected as the markers for the downstream analysis. These top ranked gene subsets denoted as ${\hat{G}}_{1}$, ${\hat{G}}_{2}$, ${\hat{G}}_{3}$, ${\hat{G}}_{4}$, ${\hat{G}}_{5}$, respectively. According to the definition, the simplified ${\underline{L}}_{i,j}$ of above signatures is consistent across multiple datasets from the corresponding tissue type and it excludes genes that do not exhibit any change between the young and old groups. We can further define a marker vector for a tissue type k as

$$M_{k}=\{{\bar{L}}_{i,j}\left|i\in {\hat{G}}_{k},j\in \mathrm{any}\;\text{datasets correspoding to}\;{\hat{G}}_{k}\right\}=\left\{{\bar{L}}_{i}|i\in {\hat{G}}_{k}\right\}$$

### 4.16 RNAge Score Calculation

The aging score is applied on a gene expression test data where two conditions are compared for expression changes in the aging signature, group1 has *n* samples and group2 has *m* samples. For each gene in a tissue specific marker set ${\hat{G}}_{k}$, we can calculate *t*‐statistic between two groups based on Welch's two‐sample *t* test as follow:

$$t_{i}=\frac{\mu_{i,\text{group}1}-\mu_{i,\text{group}2}}{\sqrt{\frac{S_{i,\text{group}1}^{2}}{n}+\frac{S_{i,\text{group}2}^{2}}{m}}}\;i\in {\hat{G}}_{k},$$

where $\mu_{i,\text{group}1}$ is the mean expression of gene i in group1, $\mu_{i,\text{group}2}$ is the mean expression of gene i in group2, $S_{i,\text{group}1}^{2}$ is the variance of gene i in group1, and $S_{i,\text{group}2}^{2}$ is the variance of gene i in group2.

All *t*‐statistics from the same tissue specific marker set ${\hat{G}}_{k}$ denoted as a vector $T_{k}=\left\{i\in {\hat{G}}_{k}\right\}$. In cases where marker gene i is not expressed in the test dataset we set $t_{i}=0$. We then defined the “RNAge score” for given tissue type k as follow:

RNAgek=TkMkNk=∑i∈G^kti×L¯iNk

where the normalizing factor $N_{k}$ is the number of overlapping genes between the test dataset and ${\hat{G}}_{k}$. The aging score, ${\text{RNAge}}_{k}$, represents the overall magnitude of expression difference between two sample groups based on the expression trends. This score is calculated using the tissue‐specific aging marker set $M_{k}$. It indicates the aging difference between the two groups as well as distinguishes which group is older. To mitigate the influence of a small number of markers with large changes gene expression between group1 and group2 of the test data (i.e., large *t*‐score values), we introduced the “Percentage score” as a measure of agreement between the expression trends of the aging markers and the given dataset. The calculation of the Percentage score involves the product of each gene between $T_{k}$ and $M_{k}$: $A_{i}=t_{i}\times {\underline{L}}_{i}$, where $i\in {\hat{G}}_{k}$. A positive $A_{i}$ indicates a consistent expression change of gene i in both the aging signature and in the comparison between group1 and group2 in the test dataset. Conversely, a negative $A_{i}$ indicates an opposite expression trend. Therefore, the “Percentage score” is defined as:

$${\text{Percentage score}}_{k}=\frac{\text{number of positive}\;A_{i}}{N_{k}},i\in {\hat{G}}_{k}$$

### 4.17 L1000 Data Analysis

The level 3 normalized expression data were downloaded from the LINCS L1000 database of cellular perturbations (Subramanian et al. 2017) (https://lincsproject.org). The perturbation data from the following cell lines were extracted for downstream analysis: 1HAE, NEU, and NPC. Replicates from each perturbation and corresponding control experiment were identified using perturbation IDs and plate IDs. Then RNAge scores were applied to above each perturbation individually. The average RNAge scores were calculated for the perturbations with the same treatment time and concentration. The top perturbation candidates were then ranked and chosen based on the RNAge subscores.

## Author Contributions

Conceptualization: C.Z., N.S., D.C., D.B., and L.S. Computational analysis: C.Z. and D.B. Lab‐based experiment: N.S. and D.C. Cell culture: N.S., A.N., T.S., S.Y.C., and A.M. Writing – original draft: N.S., C.Z., D.B., and L.S.

## Conflicts of Interest

L.S. is a scientific co‐founder and consultant of Bluerock Therapeutics and DaCapo Brainscience. All other authors declare no conflicts of interest.

## Supporting information


**Figure S1.** Derivation of RNAge from primary sequencing data. (A) Schematic outline of primary cell lines (primary fibroblasts; pFIB) and of brain tissues (frontal cortex; FC and substantia nigra; SN) sequenced in this study. Samples from young donors are in blue and old are in red. (B) PCA of RNA‐seq data from primary fibroblasts, frontal cortex, and substantia nigra from young (blue) and old (red) donors. (C) Volcano plots showing genes that are significantly differentially expressed (FDR < 0.1) between young and old primary tissues shown in (A, B). (D) Flowchart outlining the process used to select the genes used to calculate each RNAge subscore. (E) Schema outlining the steps to calculate the RNAge score. Given an aging signature (e.g., Pan‐Neuronal from (D)), we compute a *t*‐score for the expression change between two conditions (e.g., drug treatment vs. control). The *t*‐scores are transformed by a marker vector that indicates the expected directionality of aging change. The results are used to compute the aging score by simple mean and calculate the percentage of markers changing in accordance with the age signature. (F) RNAge scores of the datasets used to establish aging signature subcategories. RNA‐seq data used in Total pF, PolyA pF, Total FC, and Total SN were generated as part of this study. Gage pF and Gage FC are similar young and old samples that were profiled independently in a different study (Mertens et al. 2015). RNAge score refers to the modified *t*‐score for each pairwise comparison and is indicated by the color of the bubbles. The size of the bubbles indicates the percentage of genes that make up the aging score that change in the expected direction for young versus old. (G) Application of RNAge score to young and old human primary stomach, cerebellum, spinal cord, and hippocampus (GTEx Consortium 2013) and GBM (Ainslie et al. 2024). In all bubble plots, the primary sub scores are indicated in green text and secondary sub scores are in orange text.

**Figure S2.** Reprogramming of primary fibroblasts to iPSC and generation of iPSC‐derived fibroblasts. (A) RNA‐seq analysis showing that gene expression of fibroblast‐specific genes in iPSC‐FIB closely resembles that of pFIB, indicating the re‐establishment of fibroblast identity in iPSC‐FIB. (*N* = 6 independent cell lines at each stage; 3 derived from young donors and 3 derived from old donors). (B) Volcano plots showing DEGs (FDR < 0.1) between young and old pFibs, iPSCs derived from young and old donors, and iPSC‐FIB generated from these stem cells (*N* = 3 independent cell lines derived from young donors and *N* = 3 derived from old donors). (C) PCA of RNA‐seq data from primary fibroblasts originating from young, old, or individuals with HGPS. (D) RNAge score of young pFIBs versus HGPS pFIBs from this study data (top) and similar comparison using RNA‐seq data from Fleischer et al. (2018) (bottom) demonstrating that HGPS fibroblasts do not have an elevated RNAge score. In all bubble plots, the primary sub scores indicated in green text and secondary sub scores are in orange text.

**Figure S3.** Experimental validation of rejuvenating compound identified in silico. (A) RNAge score distribution for L1000 in silico screen performed in neural progenitor cells (NPC). (B) List of top scoring RNAge modifying compounds from (A). (C) Experimental validation of Mocetinostat as a regulator of RNAge in old primary fibroblasts. Primary sub scores are in green text and secondary sub scores are in orange text. (D) Scatter plot of the differential expression (plotted on the *y*‐axis) and differential chromatin accessibility (plotted on the *x*‐axis) between young and old primary fibroblast samples. HDAC‐i downstream targeted or associated TFs are labeled and highlighted.

**Figure S4.** Selecting the optimal concentration candidate age regulators and impact on hallmarks of aging. (A, B) Calculation of RNAge for every concentration of our candidate fibroblast (A) and neuron (B) age inducers available within the LINCS 1000 dataset. The RNAge score was calculated relative to every control sample within the dataset. Conditions with the most pronounced effect (yellow) were selected for downstream validation. (C, D) Example immunocytochemistry images of hallmarks of aging assays in fibroblasts (C) and neurons (D) after 24 h treatment with validated age inducing compounds. Scale 100 μM.

**Figure S5.** Role of fludarabine and DNA damage in regulating cellular age in vitro. (A) Quantification of the cellular hallmarks of aging in WT cortical neurons after 4 days of treatment with 1 μM fludarabine. For H3K9me3, LAP2, yH2AX, p21, BAG3, and CAV1 fluorescence intensity is relative to the DMSO control. *N* = 12; *p* values are calculated using a one‐sample *t*‐test. The experiment was repeated four times, each with three replicate wells. (B) Quantification of the cellular hallmarks of aging in WT dopaminergic neurons after 4 days of treatment with 10 μM fludarabine. For H3K9me3, LAP2, pATM, p21, BAG3, and CAV1, fluorescence intensity is relative to the DMSO control, and *p*‐values are calculated using a one‐sample *t*‐test. For nuclear area and nuclear roundness, absolute values are shown and significance testing is performed using unpaired *t*‐tests. *N* = 12; it was repeated four times, each with three replicate wells. (C) Example immunocytochemistry image showing induction of DNA damage (marked by H2A.X) in neurons in response to 24 h treatment of bleomycin (5 μM). (D) Presto blue viability assay in WT and APPswe/swe neurons treated with bleomycin (5 μM) for 4 days relative to DMSO treatment (*n* = 3).

**Table S1.** Excel file containing information regarding primary samples used in this study.

**Table S2.** Excel file containing gene expression changes in young versus old primary samples.

**Table S3.** Excel file containing the top 350 genes for each subscore. The top 100 genes were used to calculate RNAge, and the complete subset was used for pathway analysis.

**Table S4.** Excel file outlining the gene expression changes that occurred during reprogramming to pluripotency and subsequent re‐differentiation to fibroblasts.

## Data Availability

The data that support the findings of this study are openly available in NIH BioProject at https://www.ncbi.nlm.nih.gov/bioproject/PRJNA1107241/, reference number PRJNA1107241.
