# Supplementary figures and images for: Identifying Age‐Modulating Compounds Using a Novel Computational Framework for Evaluating Transcriptional Age
Source: Aging Cell. 2025 Apr 30;24(7):e70075. doi: 10.1111/acel.70075 (PMC12266757; doi:10.1111/acel.70075)

Fig. S1

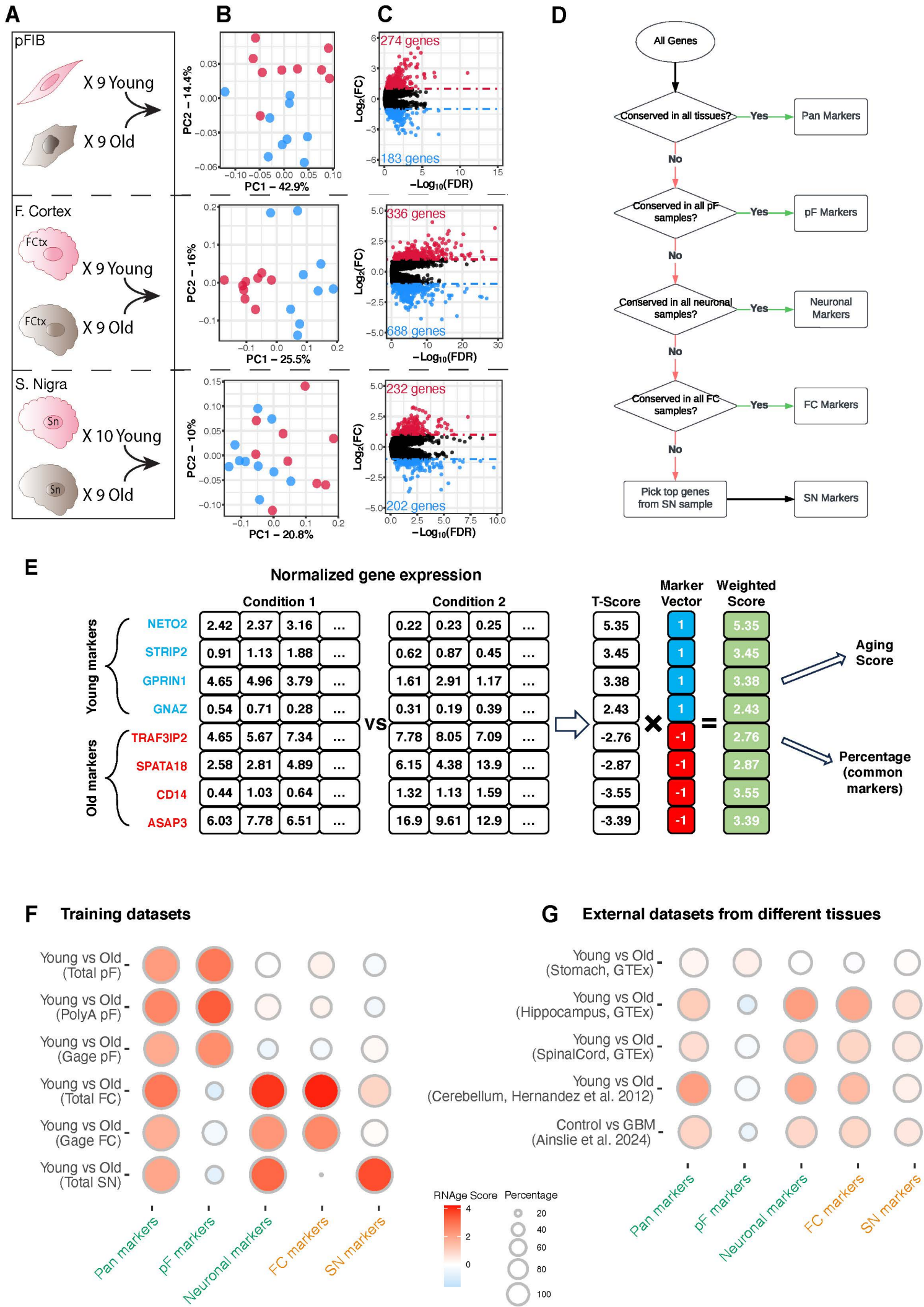

Supplement: Supplementary file 1 — Figure S1. Derivation of RNAge from primary sequencing data. (A) Schematic outline of primary cell lines (primary fibroblasts; pFIB) and of brain tissues (frontal cortex; FC and substantia nigra; SN) sequenced in this study. Samples from young donors are in blue and old are in red. (B) PCA of RNA‐seq data from primary fibroblasts, frontal cortex, and substantia nigra from young (blue) and old (red) donors. (C) Volcano plots showing genes that are significantly differentially expressed (FDR < 0.1) between young and old primary tissues shown in (A, B). (D) Flowchart outlining the process used to select the genes used to calculate each RNAge subscore. (E) Schema outlining the steps to calculate the RNAge score. Given an aging signature (e.g., Pan‐Neuronal from (D)), we compute a t‐score for the expression change between two conditions (e.g., drug treatment vs. control). The t‐scores are transformed by a marker vector that indicates the expected directionality of aging change. The results are used to compute the aging score by simple mean and calculate the percentage of markers changing in accordance with the age signature. (F) RNAge scores of the datasets used to establish aging signature subcategories. RNA‐seq data used in Total pF, PolyA pF, Total FC, and Total SN were generated as part of this study. Gage pF and Gage FC are similar young and old samples that were profiled independently in a different study (Mertens et al. 2015). RNAge score refers to the modified t‐score for each pairwise comparison and is indicated by the color of the bubbles. The size of the bubbles indicates the percentage of genes that make up the aging score that change in the expected direction for young versus old. (G) Application of RNAge score to young and old human primary stomach, cerebellum, spinal cord, and hippocampus (GTEx Consortium 2013) and GBM (Ainslie et al. 2024). In all bubble plots, the primary sub scores are indicated in green text and secondary sub scores are in orange text. [file ACEL-24-e70075-s001.pdf]

**Fig. S2**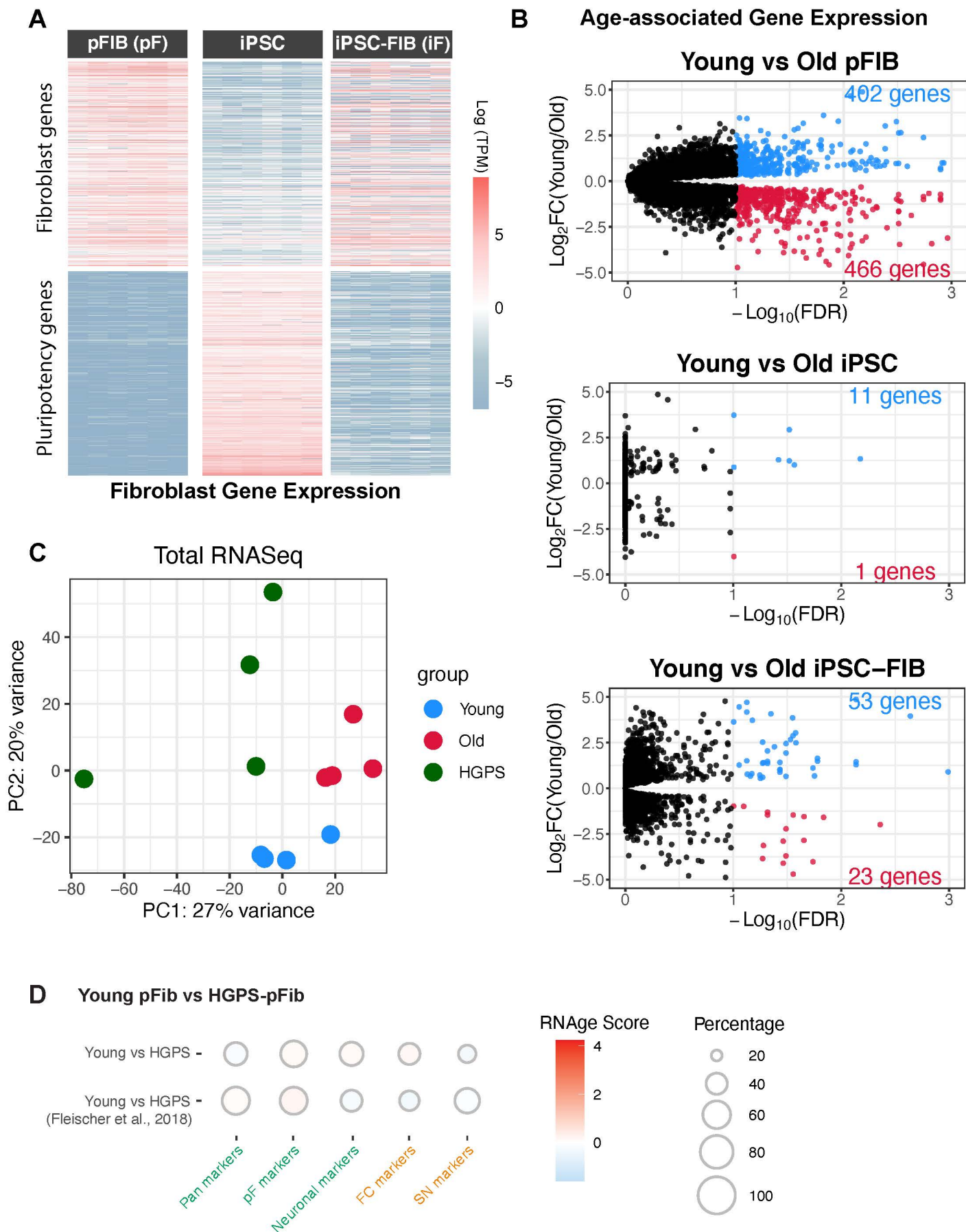

Supplement: Supplementary file 2 — Figure S2. Reprogramming of primary fibroblasts to iPSC and generation of iPSC‐derived fibroblasts. (A) RNA‐seq analysis showing that gene expression of fibroblast‐specific genes in iPSC‐FIB closely resembles that of pFIB, indicating the re‐establishment of fibroblast identity in iPSC‐FIB. (N = 6 independent cell lines at each stage; 3 derived from young donors and 3 derived from old donors). (B) Volcano plots showing DEGs (FDR < 0.1) between young and old pFibs, iPSCs derived from young and old donors, and iPSC‐FIB generated from these stem cells (N = 3 independent cell lines derived from young donors and N = 3 derived from old donors). (C) PCA of RNA‐seq data from primary fibroblasts originating from young, old, or individuals with HGPS. (D) RNAge score of young pFIBs versus HGPS pFIBs from this study data (top) and similar comparison using RNA‐seq data from Fleischer et al. (2018) (bottom) demonstrating that HGPS fibroblasts do not have an elevated RNAge score. In all bubble plots, the primary sub scores indicated in green text and secondary sub scores are in orange text. [file ACEL-24-e70075-s008.pdf]

Fig. S4

**A** Candidate aging inducers - Fibroblasts

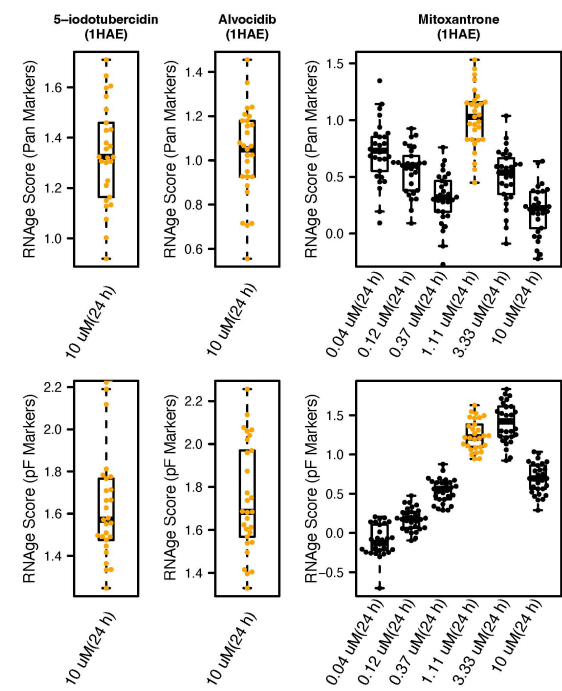

**B** Candidate aging inducers - Neurons

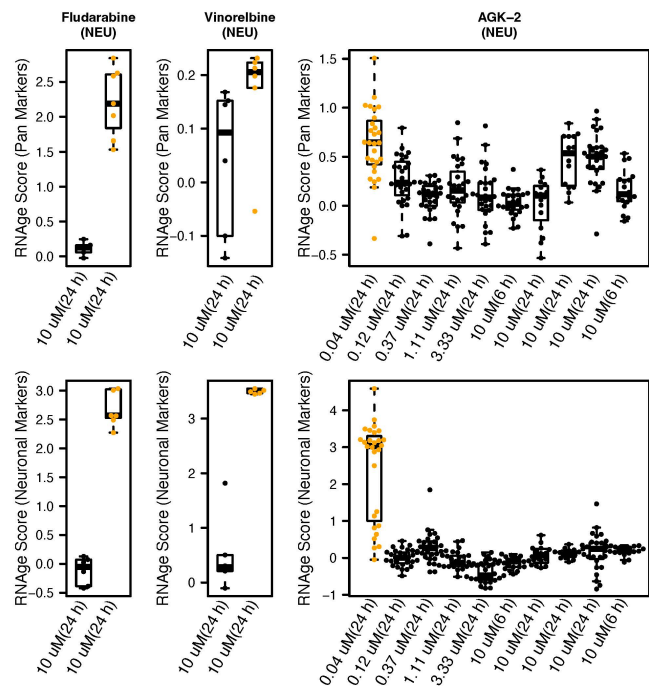

**C**

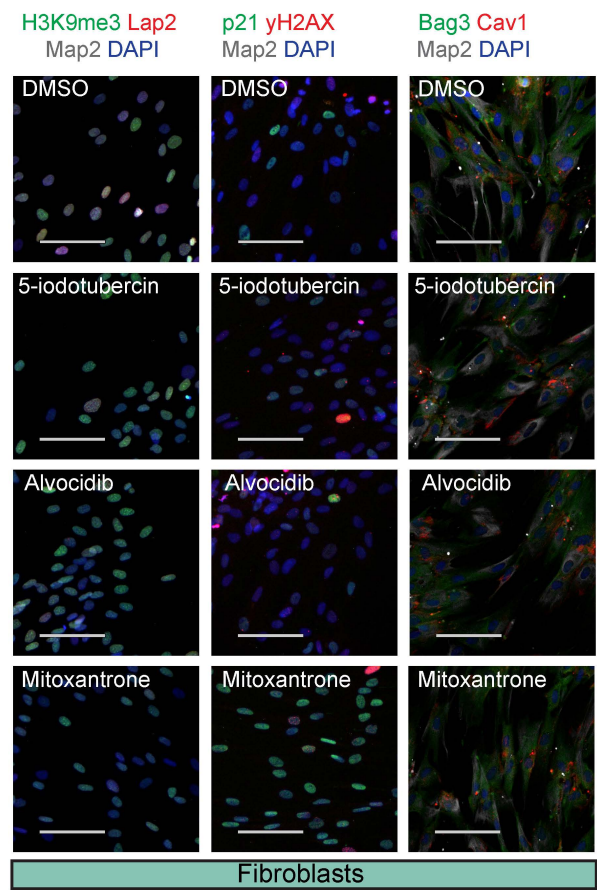

**D**

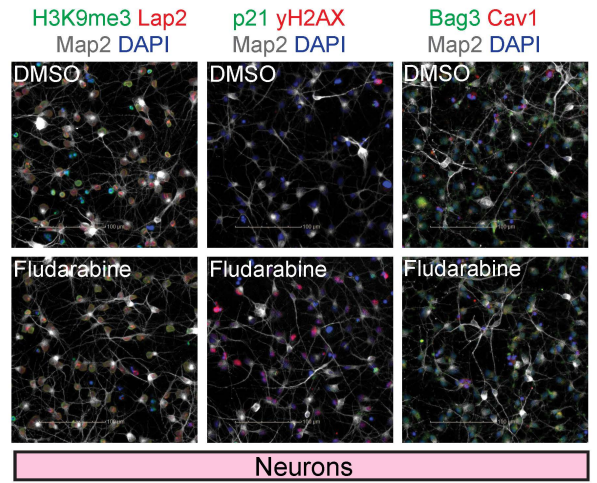

Supplement: Supplementary file 4 — Figure S4. Selecting the optimal concentration candidate age regulators and impact on hallmarks of aging. (A, B) Calculation of RNAge for every concentration of our candidate fibroblast (A) and neuron (B) age inducers available within the LINCS 1000 dataset. The RNAge score was calculated relative to every control sample within the dataset. Conditions with the most pronounced effect (yellow) were selected for downstream validation. (C, D) Example immunocytochemistry images of hallmarks of aging assays in fibroblasts (C) and neurons (D) after 24 h treatment with validated age inducing compounds. Scale 100 μM. [file ACEL-24-e70075-s007.pdf]

Fig. S5

**A Cortical Neurons: 4 day treatment**

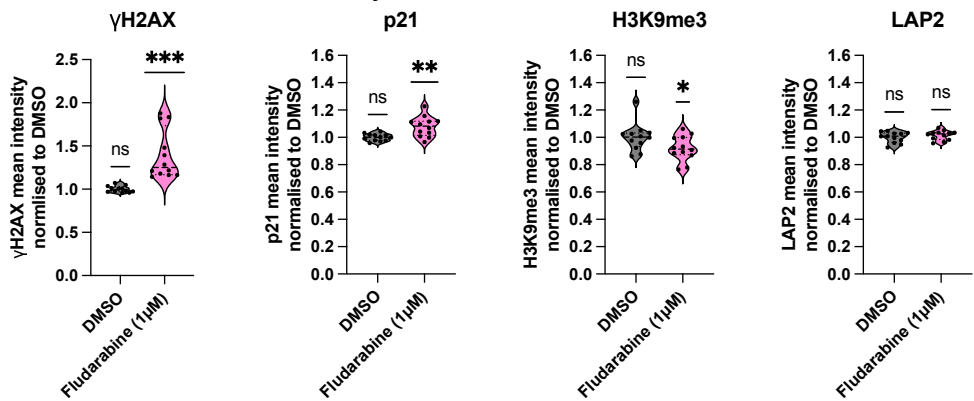

**B Dopaminergic Neurons: 4 day treatment**

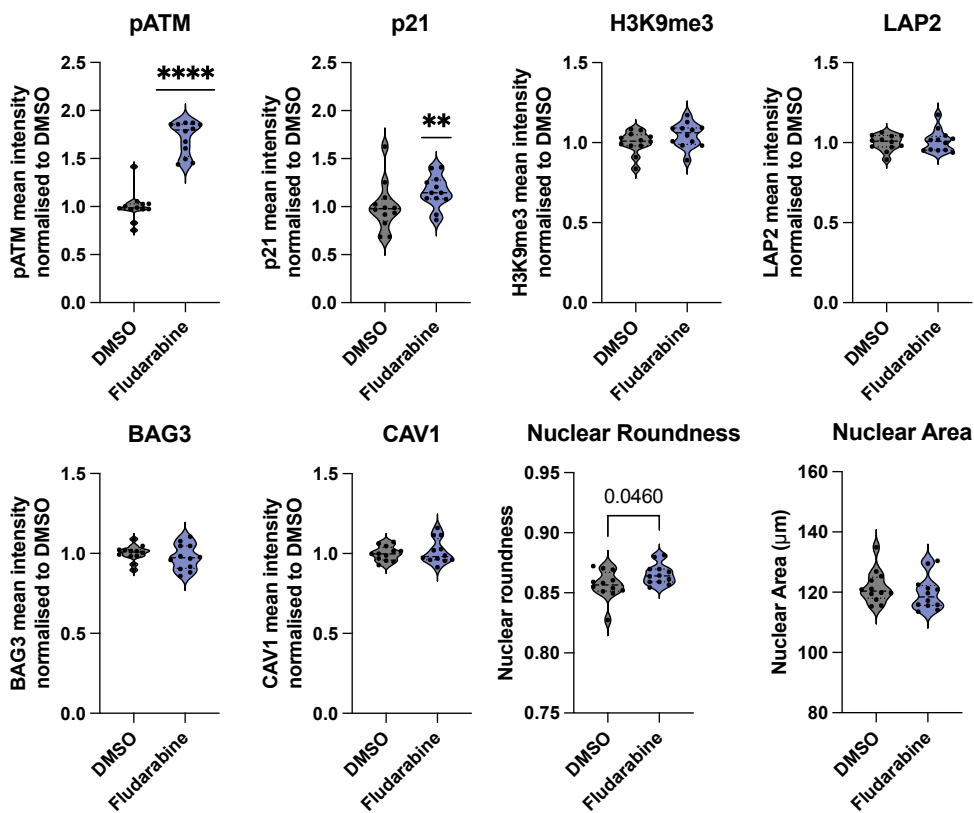

**C**

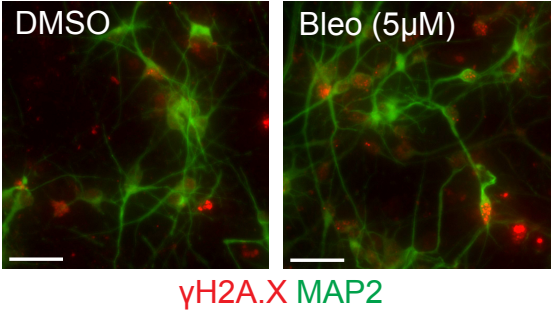

**D**

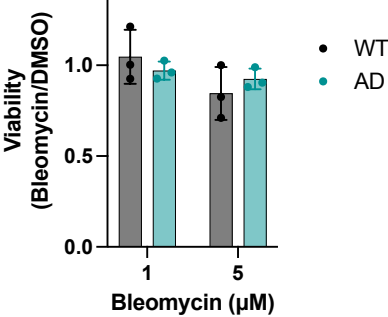

Supplement: Supplementary file 5 — Figure S5. Role of fludarabine and DNA damage in regulating cellular age in vitro. (A) Quantification of the cellular hallmarks of aging in WT cortical neurons after 4 days of treatment with 1 μM fludarabine. For H3K9me3, LAP2, yH2AX, p21, BAG3, and CAV1 fluorescence intensity is relative to the DMSO control. N = 12; p values are calculated using a one‐sample t‐test. The experiment was repeated four times, each with three replicate wells. (B) Quantification of the cellular hallmarks of aging in WT dopaminergic neurons after 4 days of treatment with 10 μM fludarabine. For H3K9me3, LAP2, pATM, p21, BAG3, and CAV1, fluorescence intensity is relative to the DMSO control, and p‐values are calculated using a one‐sample t‐test. For nuclear area and nuclear roundness, absolute values are shown and significance testing is performed using unpaired t‐tests. N = 12; it was repeated four times, each with three replicate wells. (C) Example immunocytochemistry image showing induction of DNA damage (marked by H2A.X) in neurons in response to 24 h treatment of bleomycin (5 μM). (D) Presto blue viability assay in WT and APPswe/swe neurons treated with bleomycin (5 μM) for 4 days relative to DMSO treatment (n = 3). [file ACEL-24-e70075-s002.pdf]
