# Supplementary material for: Identifying Age‐Modulating Compounds Using a Novel Computational Framework for Evaluating Transcriptional Age
Source: Aging Cell. 2025 Apr 30;24(7):e70075. doi: 10.1111/acel.70075 (PMC12266757; doi:10.1111/acel.70075)

Fig. S3

A

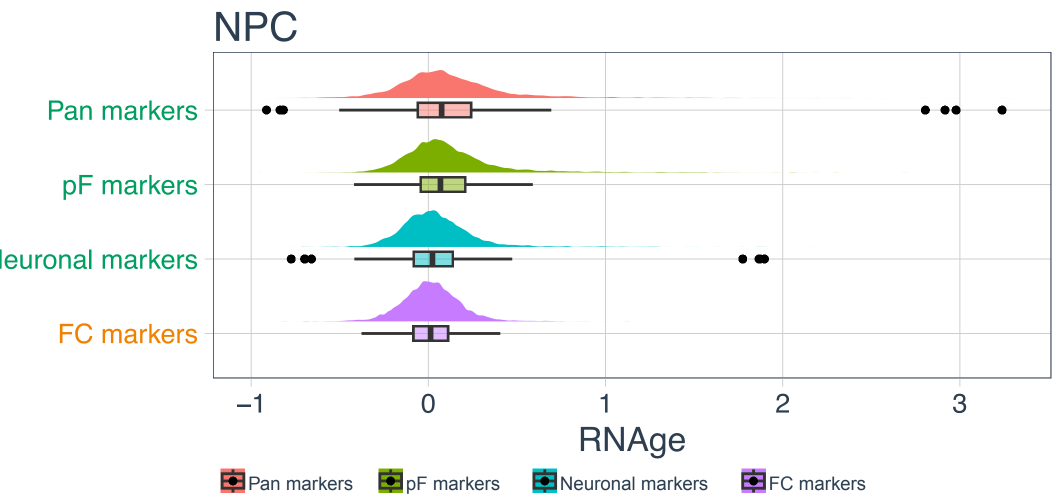

B

Compound	Score	MOA
Saracatinib	Pan, Neuronal	Src inhibitor
Mitoxantrone	Neuronal	Topoisomerase inhibitor | PKC inhibitor
Amsacrine	Pan, Neuronal	Topoisomerase inhibitor
D-4476	Pan, Neuronal	TGF beta receptor inhibitor
NSC-632839	Pan	Ubiquitin specific protease inhibitor
Mocetinostat	Neuronal	HDAC inhibitor
Entinostat	Neuronal	HDAC inhibitor
BRD-K32896438	Pan	NA
BIX-01294	Neuronal	DNMT inhibitor | HMT inhibitor
BRD-K49322988	Pan	NA

Age Inducer Rejuvinator

C

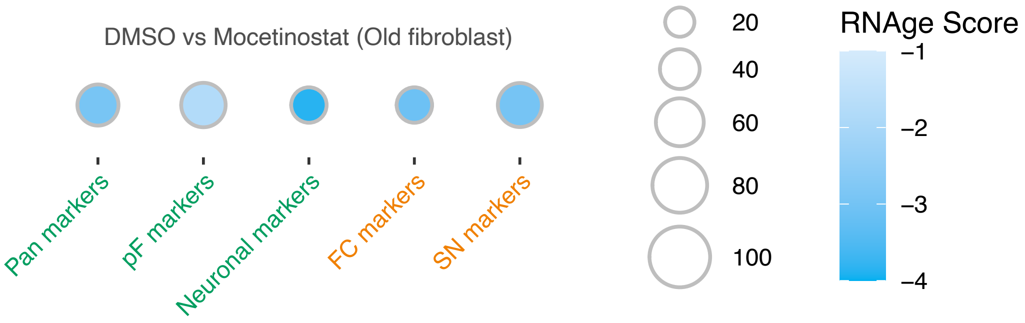

D

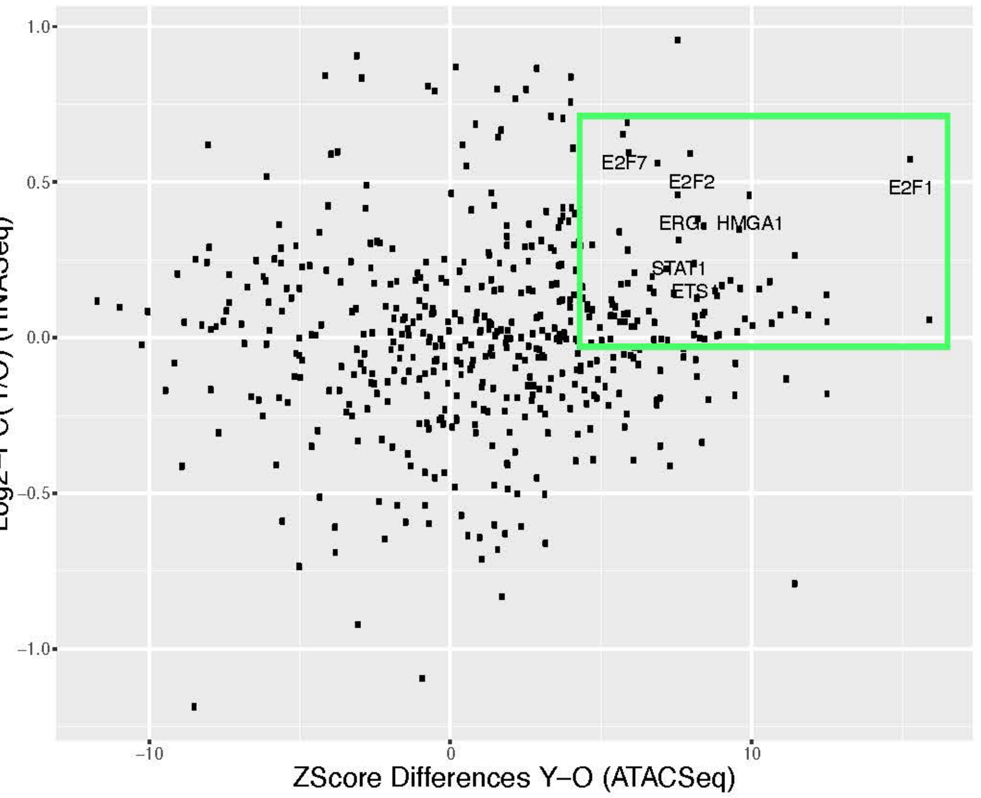

Supplement: Supplementary file 3 — Figure S3. Experimental validation of rejuvenating compound identified in silico. (A) RNAge score distribution for L1000 in silico screen performed in neural progenitor cells (NPC). (B) List of top scoring RNAge modifying compounds from (A). (C) Experimental validation of Mocetinostat as a regulator of RNAge in old primary fibroblasts. Primary sub scores are in green text and secondary sub scores are in orange text. (D) Scatter plot of the differential expression (plotted on the y‐axis) and differential chromatin accessibility (plotted on the x‐axis) between young and old primary fibroblast samples. HDAC‐i downstream targeted or associated TFs are labeled and highlighted. [file ACEL-24-e70075-s005.pdf]
